# Nucleolus assessment in invasive breast carcinoma, an objective parameter for histological grading

**DOI:** 10.55730/1300-0144.5398

**Published:** 2022-03-03

**Authors:** Seda DUMAN ÖZTÜRK, Çiğdem ÖZTÜRK, Oğuzhan OKCU, Bayram ŞEN, Recep BEDİR

**Affiliations:** 1 Department of Pathology, Recep Tayyip Erdogan University Research and Training Hospital, Rize, Turkey; 2 Department of Medical Biochemistry, Recep Tayyip Erdoğan University Research and Training Hospital, Rize, Turkey; 3 Department of Pathology, Faculty of Medicine, Recep Tayyip Erdoğan University, Rize, Turkey

**Keywords:** Breast cancers, nucleolus prominence, light microscopy

## Abstract

**Background/aim:**

The nucleolus has the potential to provide insight into how many types of cancer will progress. In this study, we examined the evaluation of the nucleolus with a microscope in widespread breast cancer tumors and whether this value contributes to tumor grading as an objective clinicopathological parameter.

**Materials and methods:**

In our study, the nucleolus was evaluated retrospectively in resections with a diagnosis of invasive breast carcinoma of the cases between January 2010 and April 2021. In total, the tumor nucleolus of 377 cases of invasive breast carcinoma was evaluated. Nucleolus evaluation was performed with light microscopy using four different modes (modified Helpap method, in 1, 5, and 10 high power fields at 40x magnification). The relationship between nucleolar scores and clinicopathological parameters was examined separately. Regrading was performed by replacing nuclear pleomorphism with the nucleolar score in the classically used histological grading system and utilizing the nucleolus score as the fourth parameter in this grading system.

**Results:**

There was no significant correlation between the prognosis of the patients and the nucleolar score. When nuclear pleomorphism and nucleolar score were replaced in the classical grading system, disease-free and overall survival were correlated with the new grading system. In addition, a relationship was found between high nucleolus score and other clinicopathological parameters (such as estrogen receptor negativity, progesterone receptor negativity, high Ki-67, triple negative, and human epidermal growth factor receptor-2 status).

**Conclusion:**

The presence of nucleolus is associated with disease-free survival and overall survival of patients, and it can be evaluated with a light microscope at no extra cost and time. Therefore, in the classical grading, using it instead of nuclear pleomorphism with low reproducibility among pathologists may provide more objective results in predicting patient prognosis.

## 1. Introduction

Breast cancer (BC) is still the most common cause of cancer death in women worldwide, despite the newly developed treatment methods and the knowledge of tumor characteristics in many respects [1]. Histological grade established by evaluating tubule formation, mitosis, and nuclear pleomorphism, is one of the most robust parameters, together with hormone receptors and axillary lymph node involvement, in directing the clinicopathological treatment of these tumors [2]. Among these three parameters, nuclear pleomorphism has a minor interobserver agreement [3,4].

In many tumors, especially the change in nucleolus size and number is considered an indicator of malignancy [5,6]. The first detailed study evaluating the relationship between the nucleolus and cancer was done by Pianese in 1896 [7]. Pianese performed a cytological analysis of many malignant tumors and observed that a particularly large nucleolus was present in the cancer cell nucleus. In the early 20th century, several studies in cancer cells confirmed the presence of very large nucleoli with broad morphological changes [8]. MacCarty also made an essential contribution to this issue by observing that “in all malignant cells, regardless of the type or origin of the neoplasm, the nucleolus is much larger than the size of the nucleus” [9]. The leading role of the nucleolus, which is closely related to cancer growth, is the synthesis of rRNA and the assembly of ribosomal subunits [10–12]. The increase in nucleolus size and number indicate a high rate of ribosome biogenesis required for cell growth and proliferation [13,14]. Changes in the nucleolus are related to epidermal growth factor, c-myc protein, and oncogene proteins, which induce cell proliferation [15,16]. In standard tissue samples, nucleoli are prominent stained by eosin in hematoxylin & eosin stained preparations due to their very high protein content [11]. Nucleolar evaluation has been performed with silver staining of argyrophilic nucleolar regulatory regions in many studies [8,17].

Nucleolus prominence in various tumors, including BC, is accepted as an indicator of poor prognosis [5,18]. Today, besides the putative clinicopathological parameters in BCs, evaluation of the nucleolus is advocated as a parameter that will provide prognostic benefit to the patients [5]. Elsharawy et al. have done the most extensive study on this subject in the literature [5]. Nuclear pleomorphism assessment, which is a parameter of the currently used grading system, differs among the observers. Therefore, in order to determine a new and more objective criterion in tumor grading, in the current study, we evaluated nucleolar prominence in invasive BCs diagnosed in our clinic. In addition, we reviewed the relationship of nucleolar prominence with prognostic and other clinicopathological parameters. Ultimately, we examined whether taking the nucleolar score as a parameter in the histological grading system would contribute to predicting the prognosis.

## 2. Materials and methods

### 2.1. Patients and tissues

We selected patients with invasive BC who were operated on in our general surgery department between January 2010 and April 2021 from our hospital database. In our study, the nucleolus was evaluated retrospectively. In total, tumor nucleolus of 377 cases of invasive breast carcinoma were evaluated. Three hundred and fifty-five of the patients were diagnosed with invasive carcinoma (invasive ductal carcinoma, invasive lobular carcinoma), and 32 of them were diagnosed with other specified histological types (tubular, medullary, papillary, etc.). Patients whose clinical data could not be accessed, who died immediately after surgery due to operative complications, were excluded from follow-up for any reason, did not have hematoxylin & eosin slides, and formalin-fixed paraffin blocks in our archive were excluded from the study.

Clinical information, including gender, age, histological tumor type, grade, tumor size, lymph node status, surgery type, and patient follow-up information, was obtained through the hospital automation system. All cases were divided into molecular subtypes according to estrogen receptor (ER), progesterone receptor (PR), human epidermal growth factor receptor-2 (HER2), and Ki-67 immunohistochemical staining patterns and histological types according to World Health Organization (WHO) BC classification [19].

As a result, 377 patients were included in the study. The nucleolus in tumor cells was evaluated with different methods, and their relationship with clinicopathological parameters, disease-free survival (DFS), and overall survival (OS) were reviewed.

Please provide concise but complete information about the materials and the analytical and statistical procedures used. This part should be as clear as possible to enable other scientists to repeat the research presented. Brand names and company locations should be supplied for all mentioned equipment, instruments, chemicals, etc.

### 2.2. Histopathological evaluation

**Figure 1:**
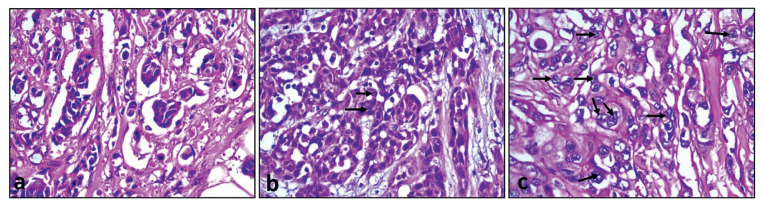
Examples of nucleolus scores 1, 2, and 3 in one high power fields. Hematoxylin & eosin x400. (a: score 1, b: score 2, c: score 3).

In this study, hematoxylin& eosin stained preparations of formaldehyde-fixed paraffin-embedded blocks (N = 377) were evaluated. Nucleolar prominence was assessed with a conventional light microscope (Olympus, BX-51, Olympus Corporation Tokyo, Japan, ocular 22mm) using four different methods as in the study of Elsharawy [5], with the modified Helpap method [20], we divided the nucleolus count into three points according to their prominence. A score of 1 was assigned to nucleolus that was not prominent in any way (i.e. inconspicuous) or nucleolus that was difficult to see at 20x magnification. Nucleolus was scored three if prominent nucleolus or dysmorphic/multiple nucleoli were present, easily seen at 10x magnification, and identified in at least 20% of the tumor. A score of 2 was assigned to nucleolus that was not evaluated as a score of 1 or 3 (Figure 1; a: score 1, b: score 2, c: score 3).

To increase objectivity, nucleolus was also scored at 40x magnification. Nucleolus in areas of one, five, and ten high power fields (HPFs) was scored as 1, 2, 3 according to the cut-off values determined (Table 1).

All evaluations were made together by two pathologists (SDÖ, ÇÖ) under a double-headed microscope. In cases where no consensus could be reached, a third pathologist’s opinion was obtained (OO).

The significance of the new histological grading (NHG1), which was formed by replacing the nuclear grade, a parameter of the histological grading used as a standard, with the nucleolus score, was examined (NHG2) (Table 2) [5].

### 2.3. Statistical analysis

Statistical analyses were performed by using IBM SPSS Statistics, Version 21.0 (IBM SPSS Statistics; IBM Corp., Armonk, NY). Descriptive statistics of the groups were given as frequencies and percentages (n, %). Before analyzing numerical variables between NHG1 and NHG2, normality analyses were performed (Kolmogorov-Smirnov and Shapiro-Wilk tests). Then, variables were reported as mean ± standard deviation or median (min–max) accordingly. The association between nucleolus score and clinicopathological variables was evaluated with the chi-square (Pearson chi-square, linear-by-linear association) and Fisher’s exact test, considering the number of patients in the categories. Prognostic factors affecting overall survival and disease-free survival were determined by univariate and multivariate Cox regression analysis. Variables with p < 0.2, which were determined by univariate analyses selected as covariates and were analyzed using the backward method. The effects of variables on survival were evaluated with the Kaplan Meier survival analysis and log-rank test. For statistical significance, the p-value was accepted as <0.05.

## 3. Results

### 3.1. Nucleolus prominence and clinicopathological parameters

Tumor nucleolus prominence was evaluated histopathologically in 377 women patients. The median age of the patients was 56 (25–100) years, and the mean follow-up period was 48 (2–136) months. Three hundred and fifty-five of the patients were diagnosed with invasive carcinoma (invasive ductal carcinoma, invasive lobular carcinoma), and 32 of them were diagnosed with other specified histological types (tubular, medullary, papillary, etc.).

According to molecular subtypes, 151 patients were evaluated as luminal A, 177 patients as luminal B, 28 patients as Her2, and 31 as triple-negative.

Evaluated with a light microscope at 20x magnification, the number of patients with nucleolus score 1 was 177, the number of patients with score 2 was 109, and the number of patients with score 3 was 101 (Table 3). There were significant differences among nucleolus scores for ER negativity, PR negativity, and high Ki-67 (p = 0.006, p = 0.042, p < 0.001, respectively). These parameters are also detailed in Table 3.

In nuclear grade evaluation simultaneously in the same patient group; there were 124 patients with grade 1, 213 patients with grade 2, and 50 patients with grade 3. The nuclear grade of the cases was primarily clustered in grade 2. When the nucleolus scoring and nuclear grade were compared, nucleolus scores were significantly concordant with nuclear grades (p < 0.001).

### 3.2. Relation of nucleolus evaluation with prognosis

**Figure 2:**
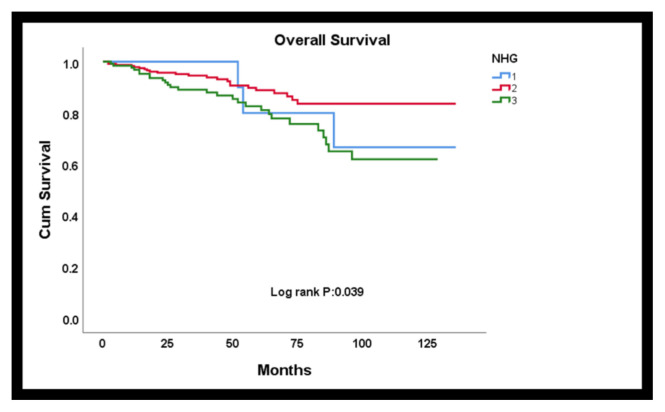
By Kaplan Meier analysis, it was found that NHG1, in which the nucleolus score and nuclear grade were replaced, was associated with overall survival.

**Figure 3:**
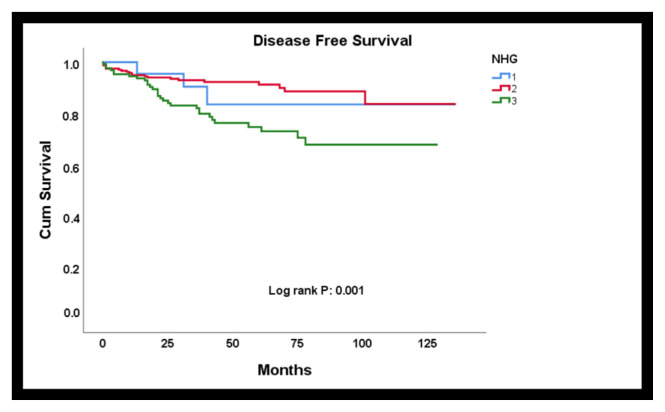
By Kaplan Meier analysis, it was found that NHG1, in which the nucleolus score and nuclear grade were replaced, was associated with disease free survival.

By Kaplan Meier analysis, it was found that NHG1, in which the nucleolus score and nuclear grade were replaced, was associated with overall survival (Log-rank p = 0.039) (Figure 2) and disease-free survival (Log-rank p = 0.001) (Figure 3). According to this, the mean life expectancy of grade 3 cases was found to be shorter than grade 1 and 2 cases (grade 3 mean survival 101.6 months). In terms of disease-free survival, similarly, high-grade patients had a worse DFS than low-grade patients (grade 3 mean disease-free survival 98.4 months).

DFS was found to be associated with NHG2, in which the nucleolus score was added as the fourth parameter (grade 3 mean disease-free survival 104.3 months). Although the mean life expectancy of grade 3 cases was shorter, it was not statistically significant (Log-rank p = 0.064).

When the relationship of the variables with OS was evaluated, in univariate analysis, metastasis, age, PR, Ki-67, molecular subtype, tumor diameter, angiolymphatic invasion, lymph node metastasis were associated with OS. In contrast, nucleolus score and nuclear grade were not associated with OS (Table 4). In multivariate analysis, molecular subtype, metastasis, age, and lymph node metastasis were determined as independent predictive variables for OS (Table 5).

When the relationship of the variables with DFS was evaluated, in the univariate analysis, molecular subtype, age, PR, Ki-67, tumor diameter, angiolymphatic invasion, lymph node metastasis, nucleolus score (10 HPFs) were associated with DFS. In multivariate analysis, Ki-67, tumor diameter, and lymph node metastasis were determined as independent predictive variables for DFS.

## 4. Discussion

Breast cancer is one of the most common malignancies among women worldwide and is the leading cause of most cancer-related deaths [21]. Despite increased early diagnosis methods and knowledge about breast cancer biology, recurrence is still seen in breast cancer cases. The histological grading system, which is formed by evaluating tubule formation, mitosis, and nuclear pleomorphism used in the routine, leads to different results among the observers, which causes different treatment methods to be applied to the patients. Helpap in 1989–1990 in renal cell carcinomas; made nucleolus grading by determining the frequency and localization of nucleolus [20]. He later used the nucleolus grading for prostate cancers as well [22].

The nucleolus, a parameter that can be evaluated by the Helpap method, is a crucial cell component that reflects the growth and increases. The increase in nucleolus prominence can be related to how much protein the cell needs. Changes in the nucleolus of cancer cells, which are very important for human life (nucleolus growth, shape differences), are indicators of poor prognosis [5,11,23,24]. Despite being so valuable, there is no consensus on the histopathological evaluation of the nucleolus in BCs. The most optimal study evaluating the nucleolus in breast carcinomas belongs to Elsharawy, K.A., and his friends [5].

In Elsharawy et al.’s study in 2020, 1200 validation, 400 training sets, and nucleolus evaluation on slides completely digitized were evaluated with four different methods: modified Helpap method, 1, 5, and 10 HPFs. The most objective method was determined as the nucleolus counted in 5 HPFs, and they showed that it was significantly associated with breast cancer-specific survival (BCSS) (p < 0.001). The high nucleolus score was associated with younger age, larger tumor size, and higher grade. They found that the inclusion of the nucleolus score in the Nottingham grading system showed a higher significant association with survival than the classical grading. This study gave rise to hope in terms of being a new parameter in grading.

Since some tumors have a heterogeneous morphology, it is necessary to evaluate different areas. For this reason, although it is more reliable to assess the entire tumor area with digitalized methods, it is not easy to reach these methods in the routine pathology practice. For this reason, while determining the nucleolus in our study, different numbers of field views were examined from the areas defined as hot spots, as in the study of Elsharawy.

In our study, nucleolus score was found to be associated with clinicopathological parameters such as ER, PR, Ki-67, molecular subtypes. Based on this, the correlation of nucleolus score with clinicopathological parameters such as ER and PR negativity, which are associated with some other poor prognosis, may be a sign of poor prognosis. However, it was not found to be related to the survival of patients alone.

Nucleolus prominence, assessed using the modified Helpap method, was reevaluated in 1, 5, and 10 HPFs. When we substituted the nuclear pleomorphism in the histological grade calculated by determining tubule formation, nuclear pleomorphism and mitosis number used in classical grading, and nucleolus scoring, a statistically significant correlation was found for OS and DFS. Survival of those with NHG 1 high grades was significantly shorter.

Nuclear pleomorphism reflects the shape, chromatin distribution, and size of the nucleolus [5]. There are no established grading criteria to evaluate nuclear pleomorphism. Therefore, this parameter, which is a very subjective criterion, leads to different results among pathologists. The minor agreement was observed for histological grade parameters among pathologists in several studies [25–28].

The lack of clear definitions and reproducibility of this grading criterion is a powerful reason to replace it with more objective components. Compared to this, nucleolus evaluation with the Modified Helpap method can be considered as a more accurate and even-handed parameter. At the same time, besides this method, nucleolus evaluation can be calculated with a light microscope with cut-off values determined with 1, 5, and 10 HPFs, while this is not the case for nuclear pleomorphism. In this sense, accepted evaluation methods for the nucleolus are relatively more objective.

OS and DFS were found to be significant with the new histological grade formed when nuclear pleomorphism and nucleolus score were displaced. Accordingly, as the new histological grade increased, OS and DFS shortened. Therefore, the nucleolus score is promising as an objective parameter of grading rather than as a stand-alone criterion.

Today, digital pathology is becoming more and more common, and we know that it has become a more objective option for pathological preparation evaluation. Elsharawy et al. also worked digitally, but this system was neither cost-effective nor practical. In our study, evaluation was made with light microscopy, and the results were found to be associated with prognosis. It is both functional and low-cost work.

There were some limitations in our article, our cases did not have a homogeneous distribution according to menopausal status, histological type, molecular subtype and age data. Some of the cases had a short follow-up period.

In conclusion, due to the low agreement among pathologists in the classical grading system, there is a need for a more objective parameter in grading. For this reason, nucleolar prominence, which can be easily and quickly evaluated with light microscopy without extra cost and time, and whose relationship with prognosis has also been proven in our study, can be used instead of nuclear pleomorphism in grading.

## Figures and Tables

**Table 1:** Cut-off points of nucleolus count suggested by Elsharawy et al [5].

Parameters	Definitions of nucleolus count		
	Score 1	Score 2	Score 3
Nucleolus count in 10 HPFs* **(at 40x)**	0–4	5–101	>101
**Nucleolus count in 5 HPFs (at 40x)**	0–2	3–50	>50
**Nucleolus count in 1 HPFs (at 40x)**	0	1–9	>9

* High power field

**Table 2:** Incorporation of nucleolar scores into the histological grading system.

Groups	Total Scores	Equivalent grade
**NHG1** * **: Grade after replacing nuclear pleomorphism score with nucleolus score**	**Total score 3, 4**	**Grade 1**
	**Total score 5, 6**	**Grade 2**
	**Total score 7, 8, 9**	**Grade 3**
**NHG2** * **: Grade after adding nucleolus score to the other three components of the grade**	**Total score 4, 5, 6**	**Grade 1**
	**Total score 7, 8, 9**	**Grade 2**
	**Total score 10, 11, 12**	**Grade 3**

* NHG: New histological grade.

**Table 3:** The relationship between the nucleolus score (by Modified Helpap’s method) and other clinicopathological parameters.

		Nucleolus score			
		1	2	3	
		n (%)	n (%)	n (%)	p
Diagnosis	Invasive Carcinoma (IC)	159 (89.8)	101 (92.7)	95 (94.1)	0.43
	Others	18 (10.2)	8 (7.3)	6 (5.9)	
Estrogen receptor	Negative	21 (11.9)	17 (15.6)	27 (26.7)	0.006
	Positive	156 (88.1)	92 (84.4)	74 (73.3)	
Progesterone receptor	Negative	42 (23.7)	29 (26.6)	38 (37.6)	0.042
	Positive	135 (76.3)	80 (73.4)	63 (62.4)	
Her 2	0	95 (53.7)	53 (48.6)	44 (43.6)	0.311
	1	23 (13)	19 (17.4)	16 (15.8)	
	2	28 (15.8)	13 (11.9)	12 (11.9)	
	3	31 (17.5)	24 (22)	29 (28.7)	
Ki-67	Low	89 (50.3)	52 (47.7)	28 (27.7)	0.001
	High	88 (49.7)	57 (52.3)	73 (72.3)	
Molecular subtypes	Luminal A	77 (43.5)	46 (42.2)	28 (27.7)	0.021
	Luminal B	81 (45.8)	48 (44)	48 (47.5)	
	HER2	9 (5.1)	9 (8.3)	10 (9.9)	
	Triple Negative	10 (5.6)	6 (5.5)	15 (14.9)	
Nuclear grade	1	47 (26.6)	21 (19.3)	1 (1)	<0.001
	2	118 (66.7)	71 (65.1)	73 (72.3)	
	3	12 (6.8)	17 (15.6)	27 (26.7)	
Histologic grade	1	17 (9.6)	10 (9.2)	6 (5.9)	0.014
	2	141 (79.7)	82 (75.2)	68 (67.3)	
	3	19 (10.7)	17 (15.6)	27 (26.7)	
Multicentricity	Negative	152 (85.9)	90 (82.6)	91 (90.1)	0.289
	Positive	25 (14.1)	19 (17.4)	10 (9.9)	
Tumor size	T1	76 (42.9)	44 (40.4)	38 (37.6)	0.276
	T2	95 (53.7)	58 (53.2)	53 (52.5)	
	T3	6 (3.4)	7 (6.4)	10 (9.9)	
Perineural Invasion	Negative	122 (68.9)	82 (75.2)	79 (78.2)	0.205
	Positive	55 (31.1)	27 (24.8)	22 (21.8)	
Angiolymphatic Invasion	Negative	92 (52)	53 (48.6)	54 (53.5)	0.766
	Positive	85 (48)	56 (51.4)	47 (46.5)	
Lymph node	Negative	94 (53.1)	59 (54.1)	59 (58.4)	0.685
	Positive	83 (46.9)	50 (45.9)	42 (41.6)	
Metastasis	Negative	152 (85.9)	95 (87.2)	86 (85.1)	0.912
	Positive	25 (14.1)	14 (12.8)	15 (14.9)	
Death	Negative	149 (84.2)	99 (90.8)	86 (85.1)	0.263
	Positive	28 (15.8)	10 (9.2)	15 (14.9)	

**Table 4:** Risk factors associated with OS in cox regression model.

	Univariate analysis	Multivariate analysis	
	p	p	HR (95% Cl)
Molecular subtypes (triple negative vs. luminal A)	<0.001	0.002	3.904 (1.682–9.06)
Nuclear grade (3 vs. 1)	0.581		
Nucleolus score (3 vs. 1)	0.952		
Nucleolus score 1 HPF*(3 vs. 1)	0.498		
Nucleolus score 5 HPFs (3 vs. 1)	0.360		
Nucleolus score 10 HPFs (2 vs. 1)	0.009		
Age	<0.001	0.001	1.058 (1.036–1.079)
Histopathologic type	0.709		
Estrogen receptor	0.013		
Progesterone receptor	0.080		
Her 2 (3 vs. 1)	0.564		
Ki-67	0.005		
HG** (3 vs. 1)	0.175		
NHG1*** (3 vs. 1)	0.389		
Multicentricity	0.531		
Tumor size (3 vs. 1)	<0.001		
Perineural invasion	0.309		
Angiolymphatic invasion	<0.001		
Lymph node	<0.001	0.017	2.355 (1.163–4.77)
Metastasis	<0.001	<0.001	4.468 (2.466–8.095)

* HPF: High power field.

** HG: Histological grade.

*** NHG: New histological grade.

**Table 5:** Risk factors associated with DFS in cox regression model.

	Univariate	Multivariate	
	p	p	HR (95% CI)
Nuclear grade (3 vs. 1)	0.407		
Nucleolus score (3 vs 1)	0.824		
Nucleolus score 1 HPF* (3 vs. 1)	0.624		
Nucleolus score 5 HPFs (3 vs. 1)	0.645		
Nucleolus score 10 HPFs (3 vs 1)	0.688		
Age	0.061	0.068	1.018 (0.999–1.037)
Histopathologic type	0.243		
Estrogen receptor	0.138		
Progesterone receptor	0.006		
Her 2 (3 vs. 0)	0.682		
Ki-67	0.001	0.001	3.038 (1.552–5.945)
Molecular subtypes (triple negative vs. luminal A)	0.001		
HG** (3 vs. 1)	0.046		
NHG1*** (3 vs. 1)	0.279		
Multicentricity	0.095		
Tumor size (3 vs. 1)	0.004	0.028	3.059 (1.13–8.281)
Perineural invasion	0.287		
Angiolymphatic invasion	0.001		
Lymph node	0.001	0.001	3.059 (1.644–5.69)

* HPF: High power field.

** HG: Histological grade.

*** NHG: New histological grade.
